# Editorial: Insights in intensive care medicine and anesthesiology: 2022

**DOI:** 10.3389/fmed.2024.1404342

**Published:** 2024-06-18

**Authors:** Ata Murat Kaynar

**Affiliations:** School of Medicine, University of Pittsburgh, Pittsburgh, PA, United States

**Keywords:** low and middle income countries (LMIC), hypoxia, remimazolam, tracheal injury, clinical trial

We are currently in the post-COVID era in healthcare, experiencing the *Great Resignation* (https://www.bls.gov/opub/mlr/2022/article/the-great-resignation-in-perspective.htm) as healthcare workers continue to suffer the aftermath of a pandemic. The 2022 Research Topic of “*Insights in Intensive Care Medicine and Anesthesiology”* is a reflection of the resilience of our anesthesiology and intensive care scientific communities worldwide, and I want to take this opportunity to thank our colleagues who are promoting science while helping patients around the world.

In a clinical trial, Part et al. (“*Pressure changes in the endotracheal tube cuff in otorhinolaryngologic surgery: a prospective observational study*”) studied the endotracheal cuff inflation pressure in patients, as this pressure is critical for effective mechanical ventilation, while higher pressures could lead to mucosal ischemia. In a single-center observational study, alterations in cuff pressure outside the appropriate range occurred in 96.0% of the patients, and 34.2% of them demonstrated inadequate endotracheal tube cuff pressure for more than 20% of the total anesthesia time. The highest rate of inappropriate cuff pressure was observed in patients undergoing head and neck surgery, with possible causative factors including positional changes, surgical procedures, anatomical manipulation, and anesthetic procedures.

In a review, Carsetti et al. (“*Anesthetic management of patients with sepsis/septic shock”)* studied the management of sepsis during the anesthetic phase. While the intraoperative period is much shorter when compared to the length of the hospital stay for a septic patient, the intraoperative period is one of the hemodynamically most tumultuous phases, as it increases the risk of inappropriate end-organ perfusion and subsequent injury. Their review is a succinct guide for practicing anesthesiologists.

In another review, Litvinova et al. (“*Patent landscape review of non-invasive medical sensors for continuous monitoring of blood pressure and their validation in critical care practice”*) explored the use of non-invasive medical sensors for continuous monitoring of blood pressure. The emerging non-invasive monitoring techniques will change the practice of hemodynamic monitoring, leading to less common use of invasive arterial catheterization as well as decreased utilization of pulmonary artery catheters.

In a clinical trial, Zhang et al. (“*Effects of opioid-free propofol or remimazolam balanced anesthesia on hypoxemia incidence in patients with obesity during gastrointestinal endoscopy: a prospective, randomized clinical trial”***)** compared propofol plus esketamine vs. remimazolam plus esketamine opioid-free balanced anesthesia on the incidence and severity of hypoxemia among obese patients undergoing gastrointestinal endoscopy. This particular group has a higher risk of intraoperative hypoxemia with associated morbidity and mortality.

Among the 264 patients in this trial, the incidence of severe hypoxemia in the remimazolam group was significantly lower than that in the propofol group. The time to recover from anesthesia was also faster in the remimazolam group, suggesting a new option for a safer anesthetic plan.

In a systematic review, Molla et al. (“*Magnitude of pediatric mortality and determinant factors in intensive care units of a low-resource country, Ethiopia: A systematic review and meta-analysis”*) studied pediatric intensive care unit (ICU) mortality in Ethiopia as compared to higher-income countries.

Multiple reports suggest an ~50% decrease in in-hospital mortality among critically ill children in the United States and Europe in the past 20 years to ~2%−4%, mainly due to centralization and a dedicated transport system, changes in admission criteria to allow less severe cases in ICUs, and changes in end-of-life care to support dying at home rather than in hospitals (1, 2). Similarly, in Korea, the mortality rates are also approximately 4.4% (3).

However, the mortality rates in Ethiopia were ~28.5%, caused by risk factors such as the use of mechanical ventilators, a Glasgow coma scale score of < 8, the presence of comorbidities, and the use of inotropes. This study highlighted the gap in healthcare in different parts of the world, stressing the need for researchers and clinicians to act.

Similar to the study by Molla et al., Endeshaw et al. (“*Survival status and predictors of mortality among patients admitted to surgical intensive care units of Addis Ababa governmental hospitals, Ethiopia: A multicenter retrospective cohort study*”) examined ICU mortality in Ethiopia in a retrospective trial. The overall ICU mortality among 378 patients over a 5-day course was staggering at 44.97%, with an incidence rate of 5.9 cases per 100 person-day observation. Trauma, a Glasgow coma scale score of < 9, readmission to the surgical intensive care unit (SICU), mechanical ventilation, and creatinine levels were found to be significantly associated with mortality in the SICU.

Both the mentioned studies underline the great disparities between low- and middle-income countries as compared to others in terms of ICU outcomes, stressing the importance of defining epidemiology so that the scientific community can act upon it to improve outcomes.

In a clinical trial, Manouchehrian et al. (“*Comparison of the effects of spinal anesthesia, paracervical block and general anesthesia on pain, nausea, vomiting and analgesic requirements in diagnostic hysteroscopy: a non-randomized clinical trial study”*) compared the effects of spinal anesthesia, paracervical block, and general anesthesia on pain; the frequency of nausea and vomiting; and the analgesic requirements in diagnostic hysteroscopy. The mean pain score during recovery and the need for analgesic injections were significantly higher in the general anesthesia group; however, no statistically significant difference was observed between the paracervical and spinal anesthesia groups. Despite reduced pain during recovery in patients receiving spinal anesthesia, the duration of anesthesia, recovery period, and return of motor function were significantly prolonged compared to those patients receiving paracervical block or general anesthesia.

Finally, in a retrospective observational study, Xu et al. (“*Association Between First 24-H Mean Body Temperature And Mortality In Patients With Diastolic Heart Failure In Intensive Care Unit: A Retrospective Cohort Study”*) studied the relationship between body temperature and mortality in patients with diastolic heart failure (DHF) in the ICU. They analyzed the Medical Information Mart for Intensive Care (MIMIC)-IV dataset and found that among 4,153 patients with DHF, body temperature (hypothermia BT < 36.5°C vs. normal 36.5°C ≤ BT < 37.5°C vs. hyperthermia BT≥37.5°C) as a continuous variable was associated with a 19% decrease in 28-day ICU mortality and with a 20% decrease in in-hospital mortality for each 1°C increase in body temperature. When temperature was used as a categorical variable, hypothermia was significantly associated with 28-day ICU mortality. Hypothermia significantly increased mortality, while hyperthermia did not.

The 2022 collection represents a sample of the studies of our colleagues from all corners of the world, and I would like to stress the importance of this platform to share knowledge to further the cause of science and health.

## Author contributions

AK: Writing – original draft, Writing – review & editing.
